# Deconstructing Cross-Entropy for Probabilistic Binary Classifiers

**DOI:** 10.3390/e20030208

**Published:** 2018-03-20

**Authors:** Daniel Ramos, Javier Franco-Pedroso, Alicia Lozano-Diez, Joaquin Gonzalez-Rodriguez

**Affiliations:** AuDIaS-Audio, Data Intelligence and Speech, Escuela Politecnica Superior, Universidad Autonoma de Madrid, Calle Francisco Tomas y Valiente 11, 28049 Madrid, Spain

**Keywords:** Bayesian, cross-entropy, probabilistic, classifier, discrimination, calibration, ECE plot

## Abstract

In this work, we analyze the cross-entropy function, widely used in classifiers both as a performance measure and as an optimization objective. We contextualize cross-entropy in the light of Bayesian decision theory, the formal probabilistic framework for making decisions, and we thoroughly analyze its motivation, meaning and interpretation from an information-theoretical point of view. In this sense, this article presents several contributions: First, we explicitly analyze the contribution to cross-entropy of (i) prior knowledge; and (ii) the value of the features in the form of a likelihood ratio. Second, we introduce a decomposition of cross-entropy into two components: discrimination and calibration. This decomposition enables the measurement of different performance aspects of a classifier in a more precise way; and justifies previously reported strategies to obtain reliable probabilities by means of the calibration of the output of a discriminating classifier. Third, we give different information-theoretical interpretations of cross-entropy, which can be useful in different application scenarios, and which are related to the concept of reference probabilities. Fourth, we present an analysis tool, the Empirical Cross-Entropy (ECE) plot, a compact representation of cross-entropy and its aforementioned decomposition. We show the power of ECE plots, as compared to other classical performance representations, in two diverse experimental examples: a speaker verification system, and a forensic case where some glass findings are present.

## 1. Introduction

Probabilistic approaches for data mining, machine learning and pattern recognition have proven their effectiveness both theoretically and practically in multiple applications [1]. As a consequence, one of the classical interests among the machine learning community has been to obtain reliable probabilities from a classifier [2,3,4,5]. More recently, this interest has been related to the active field of Deep Neural Networks [6]. We will call probabilistic classifiers to those classifiers that are designed to output probabilities.

The use of probabilistic classifiers presents many advantages against more deterministic outputs (e.g., class labels or non-probabilistic scores). First, reliable probabilistic classifiers can be easily compared because their outputs lay in the same domain. Second, it is much more straightforward to combine classifiers if they can be integrated into a theoretically sound probabilistic domain [7]. Third, a probabilistic classifier can be incorporated into a more complex model considering multiple sources of information, by the use of e.g., probabilistic graphical models [8]. Finally, probabilities are interpretable, and therefore probabilistic classifiers admit interpretation in many different domains where an end-user that operates the system as a black box wants to effectively use and understand the information it gives. Moreover, probabilistic outputs of systems have proven to be useful in many other research and application areas such as clinical decision support systems [9], cognitive psychology [10,11], biometric systems [12,13,14], weather forecasting [15] and forensic science [16,17]. In all those areas, as well as it happens with classifiers in general, Bayesian decision theory [1] constitutes the formal framework to make optimal choices of courses of action.

In order to be useful, probabilities have to distinguish between different classes, a property that has been dubbed as refinement [15,18], sharpness [19] or discrimination [13,16]. However, it is not enough: probabilities must also present a good calibration [18,20]. According to this, a better-calibrated classifier would output more reliable probabilities, and this would lead to better decisions [4,13,16]. Calibration has been measured in the past by the use of proper scoring rules [18,21,22], i.e., functions that assess the performance or goodness of probabilities. One typical example of performance metrics based on proper scoring rules is the cross-entropy function, mainly due to its information-theoretical interpretation, its good mathematical behavior and its advantageous properties [13,23].

In this article, we deeply review the cross-entropy function, as a metric of performance and as a common objective function for probabilistic classifiers. In order to focus the topic, we restrict ourselves to two-class (or binary) classifiers in a Bayesian decision scenario. Then, this article contributes in several ways, summarized here:

- We analyze the contribution to cross-entropy of two sources of information: the prior knowledge about the classes, and the value of the observations expressed as a likelihood ratio. Apart from its advantages for general classifiers [13], this analysis is of particular interest in many applications such as forensic science [17,24], where prior probabilities and likelihood ratios are computed by different agents, with different responsibilities in the decision process. Moreover, this prior-dependent analysis allows system designers to work on the likelihood ratio computed by the classifier without having to be focused on particular prior probabilities or decision costs. This way of designing classifiers has been referred to as *application-independent* [12,13]. To the best of our knowledge, there are not many works in the literature offering tools to analyze the cross-entropy function under this dichotomy.
- We introduce a decomposition of cross-entropy into two additive components: discrimination loss and calibration loss, showing its information-theoretical properties. A better discrimination component of cross-entropy will indicate a classifier that separates the classes more effectively; and a better calibration component will indicate more reliable probabilities [18]. Moreover, the decomposition enables improving calibration without changing discrimination, by means of an invertible function, theoretically justifying approaches as in [2,3,5,12,13].
- We propose several interpretations of cross-entropy that offer useful communication tools to present results of systems performance to non-expert end users, which can be of great utility for many applications such as forensic science [24].
- We generalize the use of an analysis tool, the Empirical Cross-Entropy (ECE) plot, previously used in forensic science [16,25] for two-class probabilistic classifiers. We demonstrate its power in two different experimental examples.

The article is organized as follows. For the sake of clarity, in Section 2, we give some definitions and examples, and we briefly recall Bayesian decision theory. In Section 3, we review the important concept of calibration of probabilities. We define and deeply analyze cross-entropy in Section 4. Section 5 then introduces the Empirical Cross-Entropy (ECE) plot, as a compact representation to analyze cross-entropy. In Section 6, we will show the usefulness and power of the ECE plot with respect to other classical performance measures, where two classification approaches are explored: speaker verification and a forensic case involving glass findings. Finally, a discussion and some conclusions can be found in Section 7.

## 2. Review of Bayesian Decision Theory

### 2.1. Definitions, Notation and Examples

From now on, we will use capital letters to denote random variables (e.g., *X*) and small letters for particular values observed from those variables (e.g., X=x, or simply *x*). Random (multivariate) vectors will be denoted in capital bold (e.g., X) and small bold letters will denote particular vectors observed from them (e.g., X=x, or simply x). Probabilities will be referred to as P(·), and conditional probabilities as P(·|·). Probability densities will be referred to as p(·), and p(·|·) if they are conditional. Alternative probabilistic assignments will use the same notation, but replacing *P* with $\tilde{P}$, *p* with $\tilde{p}$, and so forth. Related to this notation, we may simply denote *P* and $\tilde{P}$ as different ways of assigning probabilities.

The classification problem is better illustrated by some examples:

**Example** **1.** **Speaker Verification**. In this example, the task is to decide whether a speaker is or not a genuine user of a given access control system. Imagine that a person wants to access a resource from a bank (it could be a bank account, or simply some personalized publicity). The telephone application of the bank has an authentication facility that includes voice biometrics. In order to decide what to do with this access attempt, a classifier must compare the speech of that person with some speech that has been previously stored and processed in the application, whose identity is known and claimed by the speaker. Several sources of information must be considered by the classifier:

- Speaker recognition techniques [26] are used to perform a comparison between the speech of the person who tries to access the system and the speech stored in the system for that particular identity claimed. If the system yields a higher support that the person has the claimed identity, it will increase the chances of a successful access, and vice-versa.
- In order to make the decision, the speaker verification system must take also into account the prior probability, i.e., whether it is probable or not that an impostor attempt will happen for this particular application.
- Moreover, the bank policy should evaluate the consequences of an error when it occurs. Perhaps the application gives access to very sensitive information (e.g., operations with bank accounts), and then avoiding false attempts is critical, or, perhaps, the application yields access to personalized publicity for that particular client, and false rejections are to be avoided.

**Example** **2.** ***Forensic case involving glass findings**. In this example, imagine the role of a trier of fact (a judge or a jury) in a legal trial. There is a suspect that is accused of some crime, and it is necessary to evaluate whether the suspect was involved in a burglary. Findings related to that question include some glass pieces found in the suspect’s clothes, which could come from the window broken at the scene of the crime during the burglary. In order to evaluate those findings, the trier of fact considers two possible hypotheses: on the one hand, the glass found in the suspect’s clothes and the glass in the window of the scene of the crime comes from the same source. On the other hand, they come from different sources (although it is not always the case, for simplicity, we will assume that the trier of fact establishes that any other hypothesis is so unlikely that its probability is negligible. Moreover, these hypotheses are known as* source-generic*, and they are simpler to understand, although other more complex hypotheses are much more typical in a forensic case. However, we believe that these two assumed simplifications will help in understanding the proposed approach). An ideal trier of fact, i.e., one who correctly uses Bayesian decision theory, should consider the following:*

- The report of a forensic examiner, who compares the glass in the burglary window with the glass in the clothes of the suspect. She or he uses analytical chemistry techniques for this, as well as forensic statistic models, in order to assign a quantitative value to those findings in the form of a likelihood ratio, according to recommendations from forensic institutions worldwide [24].
- In order to make the decision, the trier of fact must also take into account the prior probability that the glass in the suspect’s clothes could be originated by the window at the scene of the crime, even before the findings are analyzed by the forensic examiner. Perhaps the suspect was arrested next to the house at the time of the burglary, and/or witnesses have seen her or him smashing the window, and/or she or he has been typically arrested in the past for similar crimes, etc., in which case the prior probability should increase. However, the suspect could present a convincing alibi, and/or witnesses could testify that they saw other people smashing the window, etc., and in those cases the prior probability should be low.
- Moreover, justice systems around the globe have legal standards and policies that influence decisions of triers of fact. In modern, advanced democracies, it is typical that a presumption of innocence is always respected, meaning that condemning a person must be supported by solid evidence. In this sense, it is naturally much more critical not to imprison an innocent person, even though this means that false acquittals may then be more probable. Thus, a trier of fact will only condemn a suspect if the probability that the suspect committed the crime is very high.

Here, we define the elements of a classification problem, contextualizing them into the described examples (speaker verification, and forensic case involving glass findings).

- Classification categories will be referred to as $\theta_{1}$ and $\theta_{2}$, and they will be assumed to be observed from a random variable Θ. It is assumed that both classes are complementary in probabilistic terms.
  – In the speaker verification example, $\theta_{1}$ stands for *the person accessing is who she or he claims to be*, and $\theta_{2}$ stands for *the person accessing is an impostor*.
  – In the forensic case example, $\theta_{1}$ stands for *the glass of the suspect’s clothes comes from the window at the crime scene*, and $\theta_{2}$ stands for *the glass of the suspect’s clothes does not come from the window at the crime scene*.
- The features observed in the classification problem will be assumed to be multivariate, and referred to as x generated by X.
  – In the speaker verification example, $x\equiv \left\{x_{a},x_{id}\right\}$, where $x_{a}$ are the speech features extracted from the utterance spoken by the person attempting to enter the system, and $x_{id}$ are the features already stored in the system, which are known to come from the claimed identity.
  – In the forensic case example, $x\equiv \left\{x_{r},x_{c}\right\}$, where $x_{r}$ are the chemical features extracted from the glass fragments *recovered* in the suspect’s clothes, and $x_{c}$ are the chemical features extracted from the window at the scene of the crime, known as *control* glass.
- The action of deciding $\theta_{i}$ will be denoted as $\alpha_{i}$.
  – In the speaker verification example, $\alpha_{1}$ means that the system decides to accept the speaker, and $\alpha_{2}$ means deciding to reject the speaker.
  – In the forensic case example, $\alpha_{1}$ means that the trier of fact decides that the suspect glass came from the window at the scene of the crime, and $\alpha_{2}$ means deciding that the suspect glass came from a different source than the window at the crime scene.
- Decision costs will be referred to as $C(\alpha_{i},\theta_{j}),$ where $\alpha_{i}$ is the decision made, but $\theta_{j}$ is the actual category to which a particular feature vector x belongs. Without loss of generality, costs are assumed to be non-negative. In addition, it is typically assumed that $C(\alpha_{i},\theta_{i})=0$, i.e., right decisions are costless.
  – In the speaker verification example, the costs are defined depending on the risk policy of the bank, and depending on the application. For instance, if we talk about access to personalized publicity, perhaps $C(\alpha_{1},\theta_{2})=1$ and $C(\alpha_{2},\theta_{1})=10$, being then flexible with false acceptances, but rigorous with false rejections. On the other hand, if the application involves accessing sensitive bank account data, perhaps the priority is to avoid false acceptances, and therefore we can set $C(\alpha_{1},\theta_{2})=50$ and $C(\alpha_{2},\theta_{1})=1$, for example.
  – In the forensic case example, costs are typically designed to avoid false condemns, even though it means that false acquittals are more frequent. Thus, a possible selection of decision costs could be $C(\alpha_{1},\theta_{2})=100$ and $C(\alpha_{2},\theta_{1})=1$. In any case, it would correspond to the trier of fact to establish the values of the costs.

### 2.2. Optimal Bayesian Decisions

Bayesian decision theory leads to a well-known decision rule, expressed as:

(1) $$\mathrm{Decide}\;\alpha_{1}\;\mathrm{iff}:\;LR_{\left(2\right)}^{\left(1\right)}>\frac{C\left(\alpha_{1},\theta_{2}\right)}{C\left(\alpha_{2},\theta_{1}\right)}\frac{P\left(\theta_{2}\right)}{P\left(\theta_{1}\right)}=\tau_{B},\;\mathrm{otherwise}\;\mathrm{decide}\;\alpha_{2},$$

where $\tau_{B}$ is the so-called Bayes threshold, and the likelihood ratio (LR) is defined as:

(2) $$LR_{\left(2\right)}^{\left(1\right)}=\frac{p\left(x\left|\theta_{1}\right.\right)}{p\left(x\left|\theta_{2}\right.\right)}.$$

Equation (1) gives the general rule to make decisions if the following quantities are known:

- The likelihood ratio $LR_{\left(2\right)}^{\left(1\right)}$, expressing the value of the observation of x in support of each of the two classes $\theta_{1}$ and $\theta_{2}$.
- The prior probabilities for both classes, namely $P\left(\theta_{1}\right)$ and $P\left(\theta_{2}\right)$.
- The costs associated to each action $\alpha_{i}$ towards deciding a class $\theta_{i}$, given that class $\theta_{j}$ is actually true, namely $C\left(\alpha_{i},\theta_{j}\right)\forall i\neq j$.

**Figure 1:**
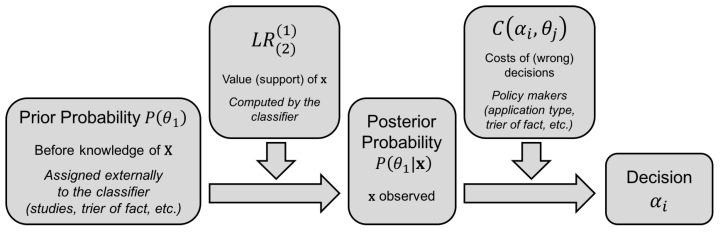
Decision scheme of a probabilistic classifier.

Figure 1 illustrates this decision scheme graphically.

Additional insights can be obtained from the so-called *odds form* of the Bayes theorem:

(3) $$\frac{P\left(\theta_{1}x\right)}{P\left(\theta_{2}x\right)}=LR_{\left(2\right)}^{\left(1\right)}\cdot \frac{P\left(\theta_{1}\right)}{P\left(\theta_{2}\right)},$$

where the odds are defined as the quotient of complementary probabilities:

(4) $$O\left(\cdot \right)=\frac{P\left(\cdot \right)}{1-P\left(\cdot \right)}$$

and the following relation can be easily derived:

(5) $$P\left(\left.\theta_{1}\right|x\right)=\frac{LR_{\left(2\right)}^{\left(1\right)}\cdot O\left(\theta_{1}\right)}{1+LR_{\left(2\right)}^{\left(1\right)}\cdot O\left(\theta_{1}\right)}.$$

Moreover, we define $\left|\log(LR_{\left(2\right)}^{\left(1\right)})\right|$ as the *strength* of the support of the features (unless explicitly stated otherwise, logarithms are assumed to be natural, i.e., base-e).

### 2.3. Empirical Performance of Probabilistic Classifiers

Empirical performance measurement involves the use of a database where ground-truth labels are available. For instance:

- In speaker verification, imagine that we have a black-box speaker recognition system that receives two sets of speech files, where all utterances in each of the two sets belong to a given putative speaker. The system compares both sets and outputs a likelihood ratio $LR_{\left(2\right)}^{\left(1\right)}$, for $\theta_{1}$ (same speaker), and $\theta_{2}$ (different speakers). With a database of speech utterances, each one with a label indicating an index of speaker identity, we compare them according to a given protocol to generate LR values. The ground-truth labels of the experimental set of LR values are same-speaker ($\Theta =\theta_{1}$) or different-speakers ($\Theta =\theta_{2}$) labels.
- In forensic interpretation of glass samples, we can work analogously with a black-box glass interpretation model yielding likelihood ratios, and a database of feature vectors measured from glass objects, each vector with a label indicating a different index for each glass object. Thus, LR values generated are accompanied by ground-truth labels like same-source ($\Theta =\theta_{1}$) and different-source ($\Theta =\theta_{2}$).

The performance of such experimental set of LR values (with ground-truth labels) can then be measured as an expected cost:

(6) $$\begin{array}{cc} E_{P\left(\alpha_{i},\theta_{j}\right)}\left[C\left(\alpha_{i},\theta_{j}\right)\right] & =P\left(\alpha_{2},\theta_{1}\right)C\left(\alpha_{2},\theta_{1}\right)+P\left(\alpha_{1},\theta_{2}\right)C\left(\alpha_{1},\theta_{2}\right)\\ & =P\left(\alpha_{2}|\theta_{1}\right)P\left(\theta_{1}\right)C\left(\alpha_{2},\theta_{1}\right)+P\left(\alpha_{1}|\theta_{2}\right)P\left(\theta_{2}\right)C\left(\alpha_{1},\theta_{2}\right), \end{array}$$

where $P\left(\alpha_{i}|\theta_{j}\right)$ is the probability of deciding action $\alpha_{i}$ when $\theta_{j}$ is the actual class of x. If i≠j, these probabilities are known as error probabilities. For our speaker recognition example, $P\left(\alpha_{1}|\theta_{2}\right)$ is known as *false acceptance probability*, and $P\left(\alpha_{2}|\theta_{1}\right)$ as *false rejection probability*. For the glass case examples, $P\left(\alpha_{1}|\theta_{2}\right)$ could be known as *false association probability*, and $P\left(\alpha_{2}|\theta_{1}\right)$ as *false exclusion probability*. Those error probabilities depend on the decision made with Bayes threshold $\tau_{B}$ (Equation (1)), for which prior probabilities and costs must be known.

## 3. Calibration of Probabilities

Bayesian decision theory is based on the fact that, for the $LR_{\left(2\right)}^{\left(1\right)}$, the probabilities $p\left(x|\theta_{1}\right)$ and $p\left(x|\theta_{2}\right)$ are known to have generated x. However, this is rarely the case for real applications, except for e.g., some simulated scenarios. Therefore, typically an alternative value ${\tilde{LR}}_{\left(2\right)}^{\left(1\right)}$ will be computed instead, as a result of directly computing the ratio in a discriminative way, or by assigning likelihoods to the data as $\tilde{p}\left(x|\theta_{1}\right)$ and $\tilde{p}\left(x|\theta_{2}\right)$. This will inevitably lead to suboptimal decisions in terms of expected cost because the decision rule in Equation (1) is assuming $LR_{\left(2\right)}^{\left(1\right)}$, not ${\tilde{LR}}_{\left(2\right)}^{\left(1\right)}$.

To address this problem, the designer of a probabilistic classifier might be tempted to keep the ${\tilde{LR}}_{\left(2\right)}^{\left(1\right)}$ values, and then to change the decision threshold τ from its theoretically optimal value $\tau_{B}$. Thus, changing the decision threshold to $\tau^{*}$ will lead to different values of $P\left(\alpha_{i}|\theta_{j}\right)$ ∀i≠j, which can optimize the expected cost in Equation (6). Of course, it might be the case that $\tau^{*}\neq \tau_{B}$.

However, our objective as designers of a probabilistic classifier should not be choosing $\tau^{*}$. For instance, in some applications, we could be even not aware of the values of the priors and decision costs, and therefore we could not compute the expected cost in order to get to $\tau^{*}$. Conversely, our aim as designers is to compute values of ${\tilde{LR}}_{\left(2\right)}^{\left(1\right)}$ that are optimal when threshold $\tau_{B}$ is set according to the values of the prior probabilities and the decision costs.

**Figure 2:**
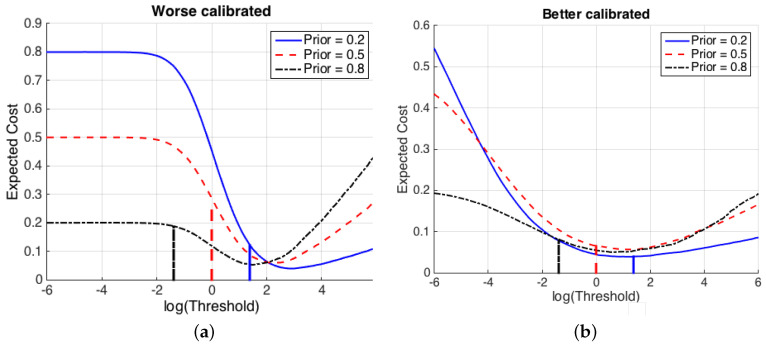
Value of the expected cost (Equation (6)) of two different sets of LR values from a speaker recognition system presenting (**a**) worse calibration (${\tilde{LR_{a}}}_{\left(2\right)}^{\left(1\right)}$ values), and (**b**) better calibration (${\tilde{LR_{b}}}_{\left(2\right)}^{\left(1\right)}$ values). Costs of erroneous decisions are set to 1 for simplicity. The *x*-axis shows possible values of a decision threshold logτ, and the logarithms of Bayes thresholds $\tau_{B}$ (Equation (1)) are shown as vertical lines.

Figure 2 illustrates the effects of a non-proper computation of ${\tilde{LR}}_{\left(2\right)}^{\left(1\right)}$ by a classifier. Two different speaker recognition systems that compute likelihood ratios for the features x are shown, and in both cases the value of the expected cost is represented for a range of thresholds $\log\left(\tau \right)$. For illustration, it is assumed that the costs of wrong decisions are both 1. Figure 2a shows a system computing ${\tilde{LR_{a}}}_{\left(2\right)}^{\left(1\right)}$ values. Bayesian thresholds ($\tau_{B}$), for each of several values of the prior probabilities, are represented as vertical lines. It is clearly observed that, for ${\tilde{LR_{a}}}_{\left(2\right)}^{\left(1\right)}$, selecting the Bayesian threshold $\tau_{B}$ leads to a suboptimal value of the expected cost for all prior probabilities. However, Figure 2b shows a system where ${\tilde{LR_{b}}}_{\left(2\right)}^{\left(1\right)}$ values have been computed, and it is shown that $\tau_{B}$ is near the optimum for all the represented values of $P\left(\theta_{1}\right)$. This is because the calibration of the likelihood ratios computed by ${\tilde{LR_{b}}}_{\left(2\right)}^{\left(1\right)}$ is much better than for ${\tilde{LR_{a}}}_{\left(2\right)}^{\left(1\right)}$. This property of calibration is of great importance in probabilistic classifiers, and it is described below.

### Calibration and Discrimination of Posterior Probabilities

The concept of calibration of probabilities is not new in the statistics literature. In [18], it was introduced in order to evaluate so-called *forecasts*, which are indeed posterior probabilities of a given hypothesis (tomorrow, it will rain) elicited by a weather forecaster. This problem is equivalent to probabilistic binary classification as proposed here, where the analogous of the forecast is $P\left(\theta_{1}|x\right)$.

In [18,27], the accuracy of such a forecaster is assessed by means of strictly proper scoring rules. One example is the logarithmic scoring rule:

(7) $$\begin{array}{c} \theta_{1}\;\mathrm{true}:\;-\log_{2}\left(P\left(\left.\theta_{1}\right|x\right)\right),\\ \theta_{2}\;\mathrm{true}:\;-\log_{2}\left(P\left(\left.\theta_{2}\right|x\right)\right). \end{array}$$

**Figure 3:**
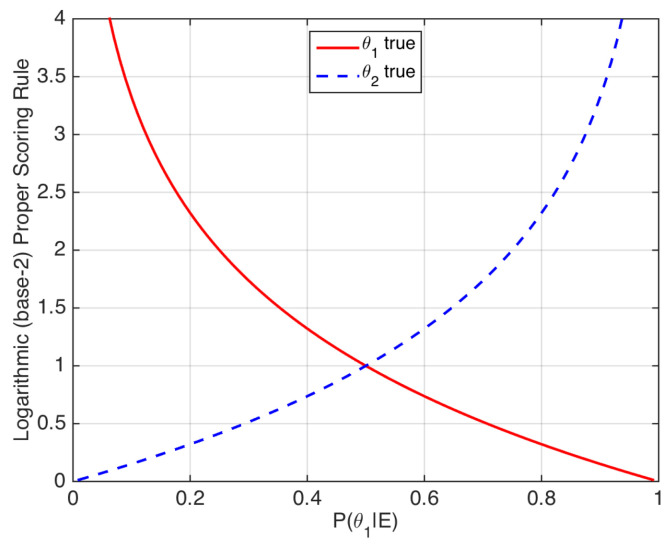
Logarithmic scoring rule. The *x*-axis represents the posterior probability of $\theta_{1}$. The rule can also be applied to prior probabilities $P\left(\theta_{1}\right)$.

Thus, strictly proper scoring rules may be seen as loss functions that assign a penalty to a given value of the posterior probability depending on the true value of the ground-truth label (see [22] for more examples of strictly proper scoring rules). The logarithmic scoring rule is illustrated in Figure 3.

Strictly proper scoring rules have the following interesting properties:

- In our speaker verification example, a posterior probability $\tilde{P}\left(\left.\theta_{1}\right|x\right)$ is obtained. We can define the mean value of the proper scoring rule with respect to a *reference* probability distribution $P\left(\left.\theta_{1}\right|x\right)$, according to [18]. This reference probability can be viewed as a desired value *P* of the posterior probability distribution, to which the actual posterior probability distribution $\tilde{P}$ is compared. For the logarithmic scoring rule:
  (8) $$-P\left(\left.\theta_{1}\right|x\right)\cdot \log_{2}\left(\tilde{P}\left(\left.\theta_{1}\right|x\right)\right)-\left(1-P\left(\left.\theta_{1}\right|x\right)\right)\cdot \log_{2}\left(1-\tilde{P}\left(\left.\theta_{1}\right|x\right)\right).$$
  By definition, the mean value of a strictly proper scoring rule is minimized if and only if $P\left(\left.\theta_{1}\right|x\right)=\tilde{P}\left(\left.\theta_{1}\right|x\right)$ (for instance, this is easy to prove for the logarithmic rule by simply deriving Equation (8) with respect to $\tilde{P}\left(\left.\theta_{1}\right|x\right)$). In other words, if a classifier aims at minimizing a strictly proper scoring rule, it should yield a likelihood ratio value as close as possible to $LR_{\left(2\right)}^{\left(1\right)}$, which will lead to the desired probability $P\left(\left.\theta_{1}\right|x\right)$.
- In [18], the overall measure of goodness of an empirical set of posterior probabilities is defined as the empirical average of a strictly proper scoring rule. An example is the logarithmic score (LS):
  (9) $$\begin{array}{ccc} LS= & - & \frac{1}{N_{1}}\sum_{x:\theta_{1}\;\mathrm{true}}\log_{2}\tilde{P}\left(\left.\theta_{1}\right|x\right)\\ & - & \frac{1}{N_{2}}\sum_{x:\theta_{2}\;\mathrm{true}}\log_{2}\tilde{P}\left(\left.\theta_{2}\right|x\right), \end{array}$$
  where $N_{1}$ and $N_{2}$ are the number of comparisons where $\theta_{1}$ or $\theta_{2}$ are respectively true. Thus, LS is an overall loss. Moreover, it is also demonstrated in [18] that such a measure of accuracy can be divided into two components:
  1. A *calibration loss* component, which measures how similar the posterior probabilities are to the frequency of occurrence of $\theta_{1}$. Low calibration loss means that, for a given range of values of $\tilde{P}\left(\left.\theta_{1}\right|x\right)$ closely around a value *k*, the frequency of cases where $\Theta =\theta_{1}$ tends to be *k*.
  2. A *refinement loss*, also known as *sharpness loss* or *discrimination loss*, component. It measures how sharp or how spread the posterior probabilities are. Roughly speaking, lower refinement loss means that, if the calibration loss is low, $\tilde{P}\left(\left.\theta_{1}\right|x\right)$ will tend to be closer either to 0 or to 1, on average.

**Figure 4:**
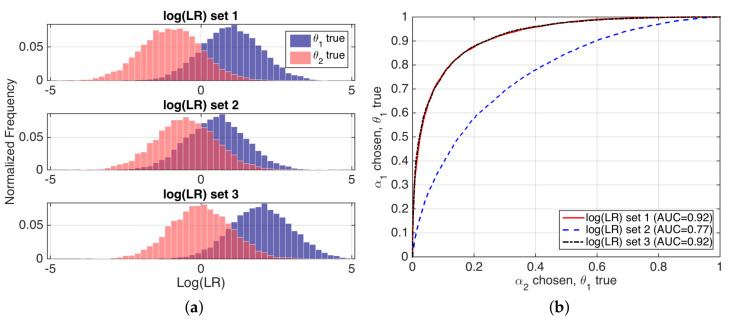
(**a**) histograms of $\log\left(LR\right)$ values for three simulated examples; and (**b**) their corresponding ROC curves, with AUC values.

Refinement loss can be seen as discrimination performance, as measured by typical performance representations such as Receiver Operation Characteristic (ROC) curves, or values such as Area Under ROC Curve (AUC). For instance, if we consider our speaker verification example, for a fixed value of the prior probabilities, the refinement, sharpness or discrimination of a set of likelihood ratios will be better if the ${\tilde{LR}}_{\left(2\right)}^{\left(1\right)}$ values of the system overlap less when $\theta_{1}$ and $\theta_{2}$ are respectively true, and so the ROC and AUC measures will be better. An illustrating example is given in Figure 4.

**Figure 5:**
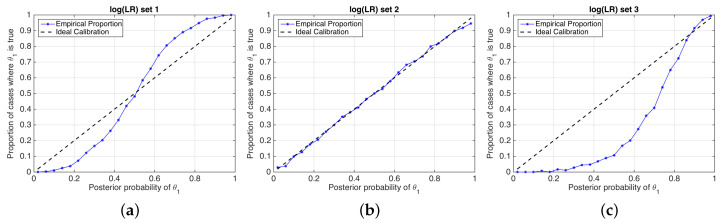
Reliability plots of the simulated sets of $\log\left(LR\right)$ values shown in Figure 4. (**a**) $\log\left(LR\right)$ set 1; (**b**) $\log\left(LR\right)$ set 2; (**c**) $\log\left(LR\right)$ set 3.

A good calibration loss means that posterior probabilities actually represent the real empirical proportion of occurrence of each hypothesis. A popular technique to measure calibration involves the use of so-called empirical calibration plots [20], also known as reliability plots [3], where the frequency of occurrence of a given hypothesis $\theta_{1}$ is represented against a binning of the posterior probabilities given by the system. Empirical calibration plots of simulated examples in Figure 4 are depicted in Figure 5.

The meaning of a good calibration loss is related to the way in which the information is presented to the decision maker, leading her or him to good decisions. This means that, if posterior probabilities are better calibrated, using the $\tau_{B}$ threshold to the likelihood ratio will yield closer-to-minimum expected cost [4,13].

## 4. Cross-Entropy: An Information-Theoretical Performance Measure

Information theory [28,29] states that the information obtained in an inferential process is determined by the reduction of the entropy, which measures the uncertainty about a given unknown variable in the light of the available knowledge. In our classification problem, the entropy represents the uncertainty that the decision maker has about the actual value of the hypothesis variable $\Theta \in \left\{\theta_{1},\theta_{2}\right\}$.

In a given classification problem, even though the features are not observed yet, the uncertainty about Θ is given by the prior probabilities $P\left(\theta_{1}\right)=1-P\left(\theta_{2}\right)$. With this available knowledge, the entropy of the hypothesis, namely prior entropy or entropy of the prior, is determined by the following expression [29]:

(10) $$\begin{array}{c} H_{P}\left(\Theta \right)=-\sum_{i\in \left\{1,2\right\}}P\left(\theta_{i}\right)\log_{2}P\left(\theta_{i}\right). \end{array}$$

Once the features x are observed in a given single classification comparison, this may or may not reduce the uncertainty about Θ. However, it can be proven [29] that the expected value of the entropy of the posterior probability over all possible values of the features x cannot be greater than the prior entropy. This expected value is a conditional entropy, which will be denoted as a posterior entropy, computed as [29]:

(11) $$\begin{array}{cc} H_{P}\left(\Theta |X\right) & =-\sum_{i\in \left\{1,2\right\}}\int_{x}p\left(\theta_{i},x\right)\log_{2}P\left(\left.\theta_{i}\right|x\right)dx \end{array}$$

(12) $$\begin{array}{c} \;=-\sum_{i\in \left\{1,2\right\}}P\left(\theta_{i}\right)\int_{x}p\left(\left.x\right|\theta_{i}\right)\log_{2}P\left(\left.\theta_{i}\right|x\right)dx, \end{array}$$

where the features x are integrated over their whole domain. As mentioned, $H_{P}\left(\Theta |X\right)\leq H_{P}\left(\Theta \right)$ [29]. However, the computation of Equation (12) is usually non-practical, as it requires knowing the likelihoods $p\left(\left.x\right|\theta_{i}\right)$, which are not known in general.

This can be solved by comparing the posterior probabilities computed by the classifier with a reference probability distribution. The letter $\tilde{P}$ ($\tilde{p}$ for pdfs) will denote probabilities obtained using the classifier, and the letter *P* (*p* for pdfs) will denote those reference probabilities. Moreover, the expression ${\tilde{LR}}_{\left(2\right)}^{\left(1\right)}$ will denote the likelihood ratio computed by the classifier, a function of x. Thus, ${\tilde{LR}}_{\left(2\right)}^{\left(1\right)}$ is the quotient between $\tilde{p}\left(\left.x\right|\theta_{1}\right)$ and $\tilde{p}\left(\left.x\right|\theta_{2}\right)$. The incorporation of the reference probabilities eliminates the dependence between $\tilde{P}\left(\left.\theta_{i}\right|x\right)$ and $p\left(x\left|\theta_{i}\right.\right)$ in Equation (12), leading to the expression of the cross-entropy:

(13) $$H_{P∥\tilde{P}}\left(\left.\Theta \right|X\right)=-\sum_{i\in \left\{1,2\right\}}P\left(\theta_{i}\right)\int_{x}p\left(x\left|\theta_{i}\right.\right)\log_{2}\tilde{P}\left(\left.\theta_{i}\right|x\right)dx.$$

It can be easily proven that the cross-entropy (Equation (13)) is decomposed into:

(14) $$H_{P∥\tilde{P}}\left(\left.\Theta \right|X\right)=H_{P}\left(\Theta |X\right)+D_{P∥\tilde{P}}\left(\left.\Theta \right|X\right),$$

where $D_{P∥\tilde{P}}\left(\left.\Theta \right|X\right)$ is the well-known Kullback–Leibler (KL) divergence between the system’s posterior distribution and the reference distribution [29] for all possible values of the evidence, defined as:

(15) $$D_{P∥\tilde{P}}\left(\left.\Theta \right|X\right)=\sum_{i\in \left\{1,2\right\}}P\left(\theta_{i}\right)\int_{x}p\left(x\left|\theta_{i}\right.\right)\log_{2}\frac{P\left(\left.\theta_{i}\right|x\right)}{\tilde{P}\left(\left.\theta_{i}\right|x\right)}dx.$$

Thus, the cross-entropy measures the complementary effect of two different magnitudes:

- $H_{P}\left(\Theta |X\right)$, the posterior entropy of the reference probability, which measures the uncertainty about the hypotheses if the reference probability distribution is used.
- $D_{P∥\tilde{P}}\left(\left.\Theta \right|X\right)$, the divergence of the classifier posterior $\tilde{P}$ from the reference posterior *P*. This is an additional information loss because it was expected that the system computed *P*, not $\tilde{P}$.

### 4.1. Proposed Measure of Accuracy: Empirical Cross-Entropy (ECE)

An empirical approximation of cross-entropy (Equation (13)) is proposed here. With an empirical set of LR values and a fixed prior probability $P\left(\theta_{1}\right)$, we can approximate Equation (13) as follows:

(16) $$\begin{array}{ccc} H_{P∥\tilde{P}}\left(\left.\Theta \right|X\right)≃ECE= & - & \frac{P\left(\theta_{1}\right)}{N_{1}}\sum_{x:\theta_{1}\;\mathrm{true}}\log_{2}\tilde{P}\left(\left.\theta_{1}\right|x\right)\\ & - & \frac{P\left(\theta_{2}\right)}{N_{2}}\sum_{x:\theta_{2}\;\mathrm{true}}\log_{2}\tilde{P}\left(\left.\theta_{2}\right|x\right), \end{array}$$

where ECE stands for Empirical Cross-Entropy. Then, by Equation (5):

(17) $$\begin{array}{c} ECE=\frac{P\left(\theta_{1}\right)}{N_{1}}\sum_{x:\theta_{1}\;\mathrm{true}}\log_{2}\left(1+\frac{1}{{\tilde{LR}}_{\left(2\right)}^{\left(1\right)}\cdot \frac{P\left(\theta_{1}\right)}{P\left(\theta_{2}\right)}}\right)\\ +\frac{P\left(\theta_{2}\right)}{N_{2}}\sum_{x:\theta_{2}\;\mathrm{true}}\log_{2}\left(1+{\tilde{LR}}_{\left(2\right)}^{\left(1\right)}\cdot \frac{P\left(\theta_{1}\right)}{P\left(\theta_{2}\right)}\right), \end{array}$$

where ${\tilde{LR}}_{\left(2\right)}^{\left(1\right)}$ is dependent of x, but we simplified the notation.

**Figure 6:**
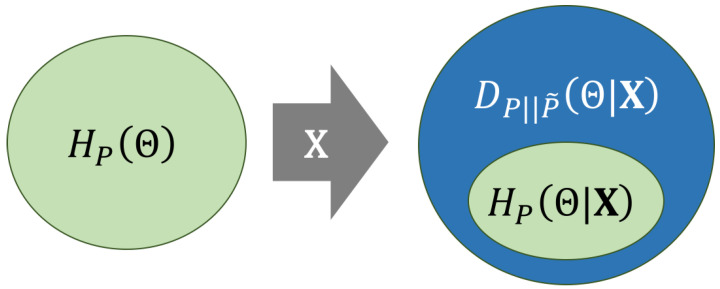
Scheme showing the decomposition of cross-entropy in Equation (14). Ellipses represent uncertainty, which can be viewed as an information loss. The ellipse on the left (green) is the prior entropy $H_{P}\left(\Theta \right)$. The ellipse on the right is the cross-entropy $H_{P∥\tilde{P}}\left(\left.\Theta \right|X\right)$ decomposed into its additive components: the posterior entropy $H_{P}\left(\Theta |X\right)$ (green) plus the KL divergence $D_{P∥\tilde{P}}\left(\left.\Theta \right|X\right)$ (blue).

Figure 6 illustrates the information loss measured by cross-entropy in terms of its decomposition (Equation (14)). There, ellipses represent uncertainty, which can be viewed also as an information loss. On the left, the prior entropy $H_{P}\left(\Theta \right)$ is represented as a green ellipse, whereas, on the right, the cross-entropy $H_{P∥\tilde{P}}\left(\left.\Theta \right|X\right)$ is represented, decomposed as $D_{P∥\tilde{P}}\left(\left.\Theta \right|X\right)$ (blue area) plus $H_{P}\left(\Theta |X\right)$ (green area). The diagram illustrates that the observation of X does not increase the uncertainty about Θ, i.e., $H_{P}\left(\Theta \right)\geq H_{P}\left(\Theta |X\right)$. In other words, we will always gain information about Θ by the observation of X, but this will only happen if reference probabilities are computed. Otherwise, the KL divergence term will contribute with an additional information loss to the cross-entropy. Thus, the cross-entropy could be arbitrarily larger than the prior entropy because $D_{P∥\tilde{P}}\left(\left.\Theta \right|X\right)$ is positive and unbounded. This could lead to a potentially dramatic loss of information if a LR model computes ${\tilde{LR}}_{\left(2\right)}^{\left(1\right)}$ values that substantially diverge from the reference $LR_{\left(2\right)}^{\left(1\right)}$.

As the prior probability is considered a parameter, then $H_{P}\left(\Theta \right)=H_{\tilde{P}}\left(\Theta \right)$. In other words, the differences between $P\left(\theta_{1}|x\right)$ and $\tilde{P}\left(\theta_{1}|x\right)$ are assumed to be only because of a different LR, not because of a different prior probability, since we want to evaluate the classifier, and the prior probability is external to it. Therefore, from Equation (17), it is straightforward that the ECE is independent of the reference likelihoods *p*. This has the following interpretation: for a fixed value of ECE, changing the reference *P* implies that:

- $H_{P}\left(\Theta |X\right)$ increases (decreases) and
- $D_{P∥\tilde{P}}\left(\left.\Theta \right|X\right)$ decreases (increases)

in order to keep ECE constant. This is depicted in Figure 6: the ellipse representing cross-entropy on the right always has the same size. However, the inner small ellipse representing posterior entropy of *P* may increase or decrease depending on the choice of the reference probability *P*.

### 4.2. Choosing a Reference Probability Distribution for Intuitive Interpretation

The selection of the reference probability *P* is constrained because Equation (14) must hold. Sensible choices of *P* should consider that, for some applications, simplicity and clarity is important, especially if one wants to communicate the results of the performance test to an end-user (like e.g., a trier of fact). In that sense, in this article, we give two proposals of reference *P*, detailed below.

#### 4.2.1. Oracle Reference $P_{o}$

The *oracle* reference distribution is motivated as follows: the aim of every classification problem is finding the true value of the hypothesis Θ. This would be achieved if the decision maker assigns the following posterior probabilities $P_{o}$:

(18) $$\begin{array}{c} P_{o}\left(\left.\theta_{1}\right|x\right)=1\;,\;\;\theta_{1}\;\mathrm{is}\;\mathrm{true},\\ P_{o}\left(\left.\theta_{1}\right|x\right)=0\;,\;\;\theta_{2}\;\mathrm{is}\;\mathrm{true}. \end{array}$$

If this oracle distribution is selected as a reference, then $H_{P_{o}}\left(\Theta |X\right)=0$, and therefore the ECE becomes solely the KL-divergence $D_{P_{o}∥\tilde{P}}\left(\left.\Theta \right|X\right)$. Thus, the higher the ECE value is, the higher the amount of information lost by the classifier in order to know the true value of the hypotheses. If the classifier does not yield oracle probabilities, then there will be an information loss. This could be also interpreted as the information needed to get to certain predictions.

#### 4.2.2. PAV-Calibrated Reference $P_{cal}$

The reference probability $P_{cal}$ will present the same discrimination loss as $\tilde{P}$ (i.e., the same ROC curve and AUC value), but it will be as perfectly calibrated as possible.

Fortunately, there is an algorithm that allows perfect calibration of probabilities, namely the Pool Adjacent Violators algorithm (PAV or PAVA) [30,31]. In fact, Ref. [32] provides a proof that PAV is the monotonic function that achieves the best possible value of a proper scoring rule in an empirical set of posterior probabilities. This also applies to ECE, as a weighted average of a proper scoring rule. The choice of $P_{cal}$ as a reference posterior distribution allows the interpretation of ECE with its two components:

- The discrimination component, namely $ECE_{min}≃H_{P_{cal}}\left(\Theta |X\right)$, that represents the information loss due to a lack of discriminating power of the classifier.
- The calibration component, namely $ECE_{cal}≃D_{P_{cal}∥\tilde{P}}\left(\left.\Theta \right|X\right)$, that represents the information loss due to a lack of calibration of the classifier.

## 5. The ECE Plot

In this paper, we propose representing ECE as a function of $P\left(\theta_{1}\right)$ in a so-called ECE plot. For each prior probability in a range centered around $P\left(\theta_{1}\right)=P\left(\theta_{2}\right)=0.5$, posterior probabilities $\tilde{P}\left(\left.\theta_{1}\right|x\right)$ are obtained using the ${\tilde{LR}}_{\left(2\right)}^{\left(1\right)}$ values computed by the classifier. The value of ECE (Equation (17)) is then represented as a function of the log-odds, namely $\log O\left(\theta_{1}\right)$. For the sake of interpretation, the *x*-axis of the ECE plots represents base-10 logarithms.

**Figure 7:**
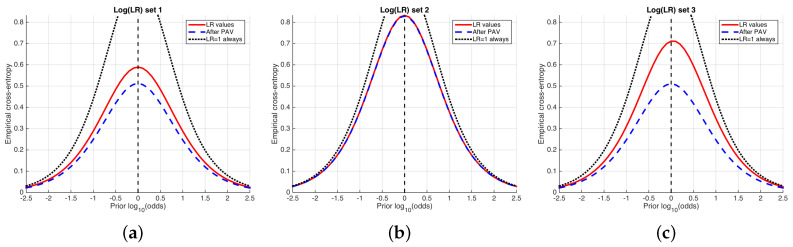
ECE plots of the simulated sets of $\log\left(LR\right)$ shown in Figure 4 and Figure 5. (**a**) $\log\left(LR\right)$ set 1; (**b**) $\log\left(LR\right)$ set 2; (**c**) $\log\left(LR\right)$ set 3.

Figure 7 shows an example of ECE plots for the simulated sets of LR values used previously in Figure 4 and Figure 5. The solid, red curve is the ECE (average information loss) of the LR values computed by the classifier. The higher this ECE curve, the higher the amount of information needed in order to know the true hypothesis, and therefore the worse the system.

Two other curves are represented in each ECE plot. On the one hand, the dashed, blue curve represents the *best-calibrated* system; for each value of the prior probability, $P_{cal}$ is obtained using the Pool Adjacent Violators (PAV) algorithm. The best-calibrated system can be seen as an optimum of performance in the sense of calibration.

On the other hand, the dotted curve represents the performance of a classifier always delivering LR=1, referred to as a *neutral* system, for which the posterior probability is equal to the prior probability (Equation (3)), and its cross-entropy is simply the prior entropy (Equation (10)). Then, if the ECE value (red, solid curve) of the classifier is greater than the entropy of the neutral system, it will be better not to use the classifier.

As a summarizing measure, ECE at the prior log-odds of 0 can be used. This measure has been already proposed as *log-likelihood-ratio cost* ($C_{llr}$) in [12,13], where its interpretation in terms of expected cost can be found (as a matter of fact, $C_{llr}$ is the expected cost of decisions, where the expectation is not only made on the action $\alpha_{i}$ and the true decision $\theta_{j}$, but also over all possible values of the costs $C\left(\alpha_{i},\theta_{j}\right)$. Thus, it is shown that optimizing the ECE value also optimizes the expected cost averaged over all values of decision costs, justifying the relationship between calibration and cost optimization as discussed in Section 3). Therefore, $C_{llr}$ can be also decomposed as ECE does, and $C_{llr}=C_{llr}^{min}+C_{llr}^{cal}$, where $C_{llr}^{min}$ and $C_{llr}^{cal}$ are, respectively, $ECE_{min}$ and $ECE_{cal}$ at the value of prior log-odds equal 0. In fact, the decomposition into discrimination and calibration by PAV was firstly proposed for $C_{llr}$ [13], and generalized to ECE afterwards [25].

Freely available Matlab${}^{TM}$ software (release 2015b, The Mathworks inc., US.) to draw ECE plots can be found in http://arantxa.ii.uam.es/~dramos/software.html.

## 6. Experimental Examples

### 6.1. Speaker Verification

Here, we test the performance of our speaker verification example in terms of ECE. We use a state-of-the-art speaker recognition system following the approach in [33]. Roughly speaking, the speaker recognition system processes the speech utterances to form feature vectors in a 600-dimensional space, the so-called i-vectors. Then, a likelihood ratio is computed by Probabilistic Linear Discriminant Analysis (PLDA), as in [34].

Supposedly, the PLDA approach should yield likelihood ratios that present good calibration, but it is not typically the case. This can be due to the high dimensionality of the problem compared to the amount of data to compare, the extreme data sparsity, and the simplicity of the model compared to the complexity of the feature space. Therefore, a further calibration transformation is typically applied to the PLDA likelihood ratio, yielding a so-called calibrated likelihood ratio. A popular calibration technique involves a logistic regression model [3,35], also known as *Platt scaling* [2].

Regarding the data used for this setup, we used the framework provided by the American National Institute of Standard and Technology (NIST) for standard benchmark tests, known as Speaker Recognition Evaluations (SRE). In our case, the empirical set used to compute likelihood ratios to test the system is the SRE 2010 condition 5 (telephone, conversational speech in English) [36]. In addition, data from previous SRE and other speech databases were used to train the PLDA model and the i-vector extractor. The equivalent condition 5 of the SRE 2008 dataset has been used to train the logistic regression model for calibration purposes [37]. We restricted the test and calibration datasets to contain only female speech. The numbers of comparisons for class is $N_{1}=3704$ and $N_{2}$ = 233,077, yielding an empirical prior proportion of $\frac{N_{1}}{N_{1}+N_{2}}≃0.016$.

**Figure 8:**
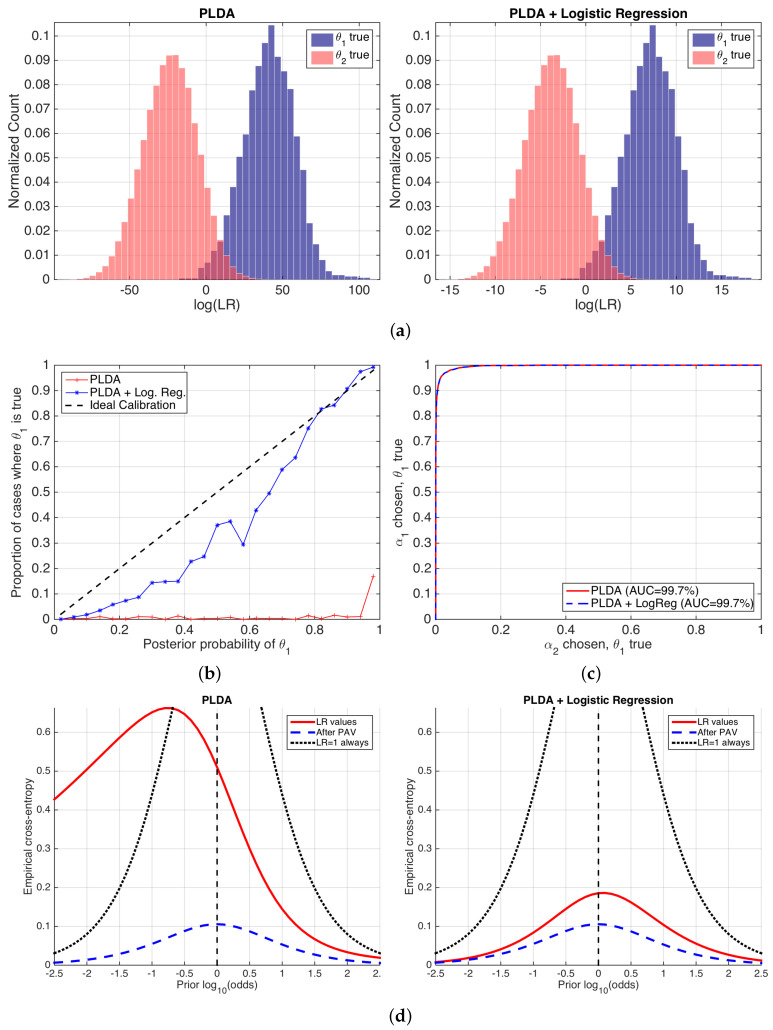
Performance of a speaker recognition system using PLDA, before and after logistic regression calibration. (**a**) histograms of $\log\left({\tilde{LR}}_{\left(2\right)}^{\left(1\right)}\right)$ values; (**b**) reliability plots; (**c**) ROC curves; and (**d**) ECE plots.

Figure 8 shows the performance of the likelihood ratios computed by the PLDA stage, before and after logistic regression calibration. We show histograms of the values of ${\tilde{LR}}_{\left(2\right)}^{\left(1\right)}$; reliability plots of $\tilde{P}\left(\theta_{1}|x\right)$ computed from ${\tilde{LR}}_{\left(2\right)}^{\left(1\right)}$ at the empirical prior $P\left(\theta_{1}\right)=0.016$; ROC curves with AUC values; and the proposed ECE plots for a wide range of prior probabilities.

Histograms in Figure 8 show equal discrimination performance because the degree of overlap between different empirical distributions is the same in both cases. This agrees with the fact that logistic regression is an invertible transformation (a sigmoid for probabilities, meaning a linear transformation for log-odds and log(LR) values), and, as a consequence, the ordering of the LR values is not altered, and therefore the discrimination performance is not changed either. However, the range of the log(LR) values is very different before and after logistic regression. This is due to the fact that the logistic regression transformation scales and shifts the histograms aiming to an improvement in the calibration of the LR values. However, the histograms after the application of logistic regression are not completely symmetric over log(LR)=0, and therefore we can expect some calibration loss. Although histograms are useful to spot tendencies in the LR values, they do not measure performance in an explicit way, and this is their main drawback as a performance representation. In reliability plots in Figure 8b, it is clear that the application of logistic regression dramatically improves calibration. As expected, the same ROC curves shown in Figure 8c indicate the same discrimination.

The previous performance measures present some problems. First, a clear decomposition between discrimination and calibration is not explicitly seen in a single figure. Although reliability plots and ROC curves separately measure both components, they are not comparable measures of discrimination and calibration loss. Finally, reliability plots only show calibration at the empirical prior.

ECE plots (Figure 8d) fix these drawbacks of previous performance representations. The performance of the set of LR values is computed for a wide range of prior log-odds (in the *x*-axis), giving a prediction of system performance for a different variety of possible applications defined by their prior probabilities. Moreover, the ECE curve is compared to the neutral curve (marked as *LR=1 always*), showing the range of prior log${}_{10}$-odds where the classifier is useless. In our example, Figure 8d shows that, for the PLDA model, the classifier will be better than doing nothing for all the range of log-odds from ca. −0.75 onwards, corresponding to $P\left(\theta_{1}\right)\geq 0.15$. However, it will be useless below this value, and end-users can be recommended not to use it in those applications. ECE plots also show that, after the application of a logistic regression stage, the classifier becomes much better because the red, solid ECE curve dramatically reduces for all the range of prior log${}_{10}$-odds analyzed. This means that the classifier will be informative for a wide range of applications. The latter is an important property, since it shows that the classifier will be useful in practically any application.

ECE plots also show the decomposition between the discrimination and calibration losses in a comparable way. The same $ECE_{min}$ curves (blue, dashed) are equal in both sets of LR values, indicating the same discrimination loss. However, the calibration loss is much higher before than after logistic regression calibration. Nevertheless, logistic regression still presents some calibration loss, explained by the dataset shift between the NIST SRE 2010 testing data, and the NIST SRE 2008 data used to train the logistic regression stage. This further motivates better ways of training the logistic regression calibration stage.

Finally, we give an example of interpretation of ECE: if we use the classifier yielding LR values by PLDA + logistic regression, and the prior probability is $P\left(\theta_{1}\right)=0.6$ for the given application (meaning prior log${}_{10}$-odds of ca. 0.8), the ECE of the classifier in that case is ca. 0.13. We can argue the following:

- We need 0.13 bits to know the true value of the ground-truth label of a comparison (i.e., we use $P_{o}$ as the reference).
- The best possible calibrated classifier needs 0.07 bits to know the true value of the ground-truth label of a comparison. However, as our classifier is not so well calibrated, it will need 0.05 bits more (i.e., we use $P_{cal}$ as reference).

In both cases, the values in bits can be also interpreted as minimum number of bits needed to describe the hypothesis variable Θ, according to information theory for data compression [29]. This has the additional insight that the higher the ECE, the more costly it is to represent the truth about the correct hypothesis, and the more information is needed to arrive at it.

### 6.2. Forensic Case Involving Glass Findings

In this section, two different models to compute likelihood ratios for glass findings are compared by ECE plots. First, a classical approach based on a multivariate model is used, with normal assumptions for the within-source variation of the features and a kernel density function used for the between-source variation, and it will be denoted as the Multivariate Kernel (MVK) model [38]. On the other hand, a model replacing the kernel density distribution with a Gaussian mixture is used, namely a Multivariate Gaussian Mixture Model (MVGMM) model [39].

A public database of glass features is used as in [38] (the dataset can be downloaded from http://onlinelibrary.wiley.com/journal/10.1111/(ISSN)1467-9876/homepage/glass-data.txt (last accessed on January 2018)). It contains features computed as log-ratios involving three combinations of chemical elements measured on glass fragments (log(Ca/K), log(Ca/Si) and log(Ca/Fe)), using some analytical chemistry technique. The dataset contains the measurements of five fragments for each of the 62 different glass objects in the database. The numbers of comparisons for each of the classes are $N_{1}=3762$ and $N_{2}=7564$, which yields an empirical prior of $\frac{N_{1}}{N_{1}+N_{2}}=\frac{1}{3}$.

**Figure 9:**
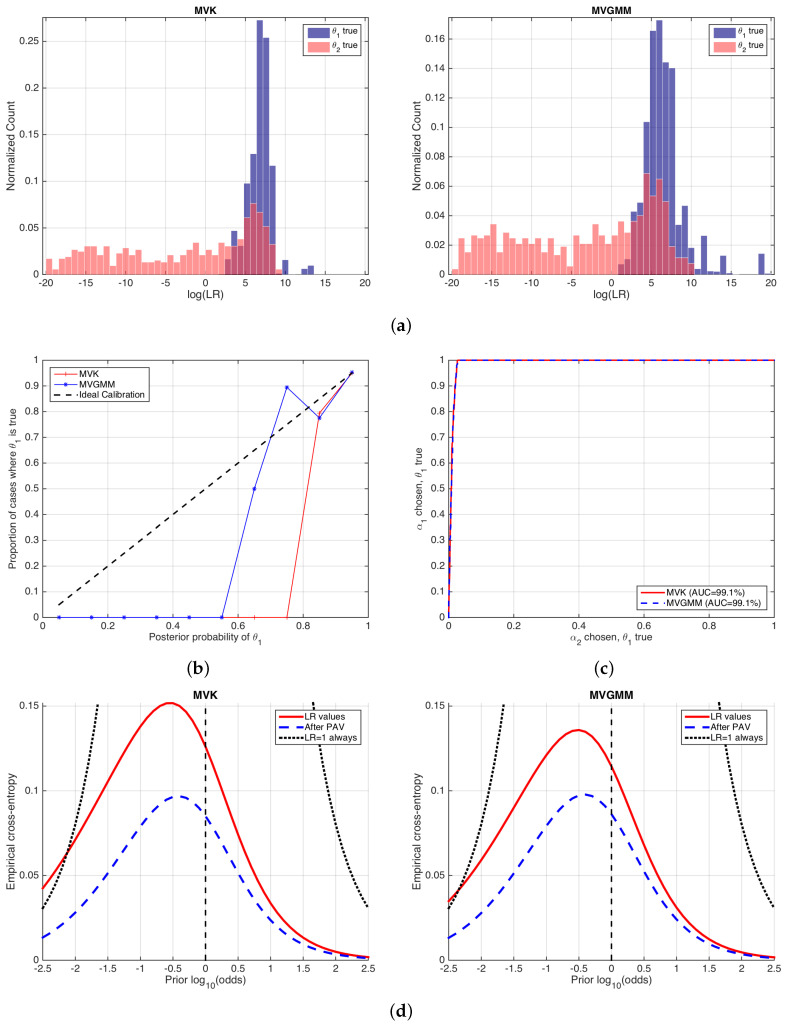
Performance of MVK and MVGMM glass evaluation models. (**a**) histograms of $\log\left({\tilde{LR}}_{\left(2\right)}^{\left(1\right)}\right)$ values; (**b**) reliability plots; (**c**) ROC curves; and (**d**) ECE plots.

Figure 9 shows the performance of the likelihood ratios computed by MVK and MVGMM. We will reason analogously as in the previous example in Section 6.1. The histograms in Figure 9a show that the $\log\left({\tilde{LR}}_{\left(2\right)}^{\left(1\right)}\right)$ values computed by both forensic glass evaluation models are similar. They show a quite asymmetric behavior, although the histograms for the MVGMM case are slightly more symmetric, which in some sense indicates a slightly better calibration performance. This is also made evident in the reliability plots in Figure 9b, where it is also seen that the calibration is quite defective in both cases. ROC curves in Figure 9c show almost the same discrimination performance.

ECE plots are shown in Figure 9d. Here, it is clear that the MVK and MVGMM models have the same discrimination loss, since the $ECE_{min}$ curves (blue, dashed) are equal. Moreover, it is seen that the calibration loss is also comparable in both approaches, since the $ECE_{cal}$ (difference between red solid and blue dashed curves) is also comparable, although slightly better in the MVGMM approach.

Here, we highlight an important fact. It can be seen that both the MVK and the MVGMM classifiers have a performance worse than the neutral curve for some range of prior log${}_{10}$-odds, namely lower than −2.5 for MVK and lower than −2.6 for MVGMM. These correspond to prior probabilities respectively of $P\left(\theta_{1}\right)\leq 0.032$ and $P\left(\theta_{1}\right)\leq 0.025$. This means that, if the trier of fact assigns a prior probability lower than those values, it is better that she or he does not make use of the report on glass analysis given by the forensic examiner because it will be misleading. This is relevant in forensic science because the forensic examiner will seldom know the prior probability assigned by the trier of fact. However, she or he can, at least, warn the trier of fact about the range of prior probabilities where the forensic report should not be considered. This property of ECE plots is definitely not given by any of the other performance representations described in this article.

Information-theoretical interpretations can be also made as in the speaker verification example, taking $P_{o}$ or $P_{cal}$ as references. However, in this case, this is even more important because, in a forensic case, end-users are typically not proficient in mathematics and statistics, and care should be taken in order to explain the performance results if requested on trial (in any case, the authors discourage the use of complex performance representations on trial given the difficulties of human beings in general and triers of fact in particular to understand forensic statistics. A recent study about this can be found in [40]).

## 7. Discussion

In our opinion, this article presents sound contributions for the general fields of pattern recognition, data mining and machine learning. One of these contributions is showing the, typically independent, sources of information affecting cross-entropy: prior knowledge and value of the features (computed as a likelihood ratio). These are not typically taken into account by previous approaches using cross-entropy in classifiers, such as in [3,5,6], where it is most common that the empirical prior probability is used solely. Other related measures such as Confusion Entropy (CEN) or Matthews Correlation Coefficient (MCC) [41,42] work with decision errors rather than probabilities, which implies the selection of a threshold τ, and therefore they do not consider performance at different prior probabilities either (moreover, in [42] it is not recommended to use CEN in two-class problems, while ECE is perfectly suited to the task). However, the importance of this prior-dependent analysis is highly relevant, since we should aim at classifiers computing LR values that can work properly for very different prior probabilities and costs, as it has been shown critical in the described examples.

Another contribution of this article is the decomposition of cross-entropy into its discrimination and calibration components. Not only can this significantly help system designers to focus on the right problem, but it also theoretically justifies the use of invertible transformations of likelihood ratios as in [3,5,13]. In forensics, this is currently a trend in many disciplines such as chemistry [43], biometrics [14], or voice comparison [44,45], as well as in some more general classification problems such as speaker verification [13]. One of the motivations of this article is precisely to foster the use of these calibration approaches for any classifier with the help of ECE plots.

The interpretations provided in terms of information theory, thanks to the use of reference probability distributions for the cross-entropy, are also an important contribution. As no *true* probability can be invoked whatsoever as the reference probability to be compared in the cross-entropy formulation, those reference probabilities should be carefully established in order to yield a useful interpretation, as it happens with oracle and calibrated references.

We strongly believe that all these contributions of the present article will help scholars and system designers in the pattern recognition, data mining and machine learning areas. We tried to provide a better understanding of the importance and usefulness of cross-entropy, in order to take advantage of its power as a performance measure in a deeper way by the use of ECE plots, and to increase its use for improving modern artificial intelligence and facing new data mining challenges.
